# Reproductive Choices and Family-building Decision Uncertainty Among Young Women at Familial Risk for Breast Cancer

**DOI:** 10.21203/rs.3.rs-10935505/v1

**Published:** 2026-10-30

**Authors:** Alexandra M. Davis, Jennifer Barsky Reese, Esther M. John, Allison W. Kurian, Jennifer S. Ford, Irene L. Andrulis, Sarah V. Colonna, Mary B. Daly, Jeanine Genkinger, Melissa C. Southey, Mary Beth Terry, Catherine Benedict

**Affiliations:** 1 PAU-Stanford Psy.D. Consortium, Palo Alto University, Palo Alto, CA, United States.; 2 Cancer Prevention and Control Program, Fox Chase Cancer Center, Philadelphia, PA, United States.; 3 Department of Epidemiology & Population Health, Stanford University School of Medicine, Stanford, CA, United States.; 4 Department of Medicine (Oncology), Stanford University School of Medicine, Stanford, CA, United States.; 5 Stanford Cancer Institute, Stanford University School of Medicine, Stanford, CA, United States.; 6 Hunter College, City University of New York, New York, NY, United States.; 7 The Graduate Center, City University of New York, New York, NY, United States.; 8 Lunenfeld-Tanenbaum Research Institute, Sinai Health System, Department of Molecular Genetics, University of Toronto, Toronto, ON M5G 1X5, Canada.; 9 Department of Internal Medicine and Huntsman Cancer Institute, University of Utah, Salt Lake City, UT, United States.; 10 Department of Clinical Genetics, Fox Chase Cancer Center, Philadelphia, PA, United States.; 11 Columbia Mailman School of Public Health, Columbia University Irving Medical Center, New York, NY, United States.; 12 Cancer Epidemiology Division, Cancer Council Victoria, East Melbourne, VIC 3002, Australia.; 13 Precision Medicine, School of Clinical Sciences at Monash Health, Monash University, Clayton, VIC 3800, Australia.; 14 Genetic Epidemiology Laboratory, Department of Pathology, The University of Melbourne, Parkville, VIC 3010, Australia.; 15 Herbert Irving Comprehensive Cancer Center, Columbia University Medical Center, New York, NY 10032, United States.; 16 Silent Spring Institute, Newton, MA, United States; 17 Department of Psychiatry & Behavioral Sciences, Stanford University School of Medicine, Stanford, CA, United States.

**Keywords:** breast cancer, family building, reproductive decisions, decision uncertainty, genetic risk, perceived knowledge, family history

## Abstract

**Background:**

A family history of breast cancer may impact reproductive choices and decisions about family-building. This study evaluated family-building considerations, plans, and decisional processes among women with a breast cancer family history, and examined sociodemographic, clinical, and psychosocial factors related to decision uncertainty.

**Methods:**

Cross-sectional data from the Breast Cancer Family Registry (BCFR) Young Women’s Study were analyzed. Participants were women (18–45 years old) with a breast cancer family history. They completed questionnaires including a Reproductive Choices module. Bivariate analyses and multiple regression evaluated relationships among study variables.

**Results:**

Participants (N=471) were on average 30.3 years old (SD=6.7), predominantly non-Hispanic White (75.1%), and over half were childless (58.6%); nearly half (47.8%) desired future children, 16.6% were unsure, and 35.7% did not. Among those interested/unsure (n=303), most considered natural conception (85.8%); many were unsure about alternative options such as embryo genetic testing, donor gametes, surrogacy, and adoption. Between 43–57% endorsed some degree of decision uncertainty. Perceived knowledge about genetic cancer risk and family-building options appeared low overall. In the final model, greater decision uncertainty was associated with having no history of pregnancy, identifying as Ashkenazi Jewish, endorsing concern about a future child’s health, lack of concern about family-building timeline, and lower perceived knowledge about genetic cancer risk and family-building options.

**Conclusions:**

Among women with a familial risk for breast cancer, decision uncertainty about family-building options is prevalent and is driven by health-related concerns, knowledge gaps, financial barriers, and values-based considerations. Findings can inform targeted decision support interventions for this population.

Up to 13% of women in the U.S. will develop invasive breast cancer at some point in their lives, and 5–10% of breast cancers are heritable [1,2]. For women of reproductive age, perceptions of cancer risk may impact the ways in which they make decisions about reproduction and plan for future family building, including the number, timing, and methods of having children [3]. Decisions may be impacted by fears stemming from personal health risks or worries about the health of a future child and passing on a genetic risk for cancer [4]. Women may face decisions about risk-reducing surgeries that impact reproductive potential and timeline and/or considerations of alternative family-building options such as through the use of assisted reproductive technology (ART), including in vitro fertilization (IVF) and preimplantation genetic testing (PGT), or adoption [5–7]. Decisions are preference-sensitive and depend on personal values, priorities, and goals. Uncertainty and distress related to family-building decisions are well documented in cancer survivor and infertility populations [8,9], but limited research has characterized the ways in which a family cancer history may impact decision making in non-clinical populations.

Among women with a high genetic risk for cancer, decision uncertainty about reproduction and family building has been associated with lower satisfaction with decision outcomes and greater likelihood of experiencing regret and animosity towards healthcare providers [10,11]. Individual-level factors such as knowledge, values, perceived risks and benefits, and access to resources and support may further shape decision making [12]. However, research has been largely limited to qualitative studies and focused exclusively on populations with a *BRCA1/2* germline pathogenic variants [13]. Most women with a family history of breast cancer do not carry a germline pathogenic variant (e.g., only 2.7% of unaffected women with at least one first-degree female relative with breast cancer [14]). Although the majority of women who undergo genetic testing due to a family cancer history will receive a negative test result, findings suggest they may still be categorized as high-risk for the development of breast cancer [15,16]. Thus, there is a strong need for quantitative evaluation of reproductive choices and family-building decisions that includes a broader population of women with a family history of breast cancer to identify unmet needs and inform clinical care and resources.

The Young Women’s Study (YWS) was designed to understand the experiences of young adult women with a family history of breast cancer – irrespective of hereditary cancer risk – across a broad range of psychosocial and medical factors and is an extension of the Breast Cancer Family Registry (BCFR) [17,18]. The BCFR cohort includes carriers of pathogenic variants in *BRCA1*, *BRCA2*, and other cancer susceptibility genes, as well as women with no identified germline variant or not tested. The current study examined reproductive choices and family-building decisional processes in the YWS cohort. The primary aims were to understand family-building plans and experience of decision uncertainty among women with a family history of breast cancer and to characterize relevant factors that influence family-building decisions in this population.

## Methods

Data collection for the YWS was completed between 2019 and 2023 across six BCFR sites: Australia (University of Melbourne), New York City (Columbia University), Northern California (Stanford University), Ontario, Canada (Lunenfeld-Tanenbaum Research Institute), Philadelphia (Fox Chase Cancer Center), and Utah (Huntsman Cancer Institute, University of Utah). The YWS was approved by the Institutional Review Boards of the participating institutions and study participants completed informed consent for future unspecified research prior to study enrollment.

### Participants

Inclusion criteria for the YWS included: assigned female at birth, age 18–45 years at the time of enrollment, and a relative of a member with breast cancer enrolled in the BCRF (i.e., with a family history of breast cancer defined as “any relative”). For the present analysis, those with a personal history of cancer were excluded. A total of 873 YWS participants completed the sociodemographics survey and 798 (91%) were between the ages of 18 and 45 years at the time of survey completion. Among those, 504 (63%) completed the Reproductive Choices module; 33 participants were excluded due to a personal history of cancer or indicating that a family history of cancer was “not applicable.” The final sample, which comprised women with data on reproductive choices, included 471 participants.

### Data Collection

YWS participants completed self-administered online questionnaire modules, including the Reproductive Choices module, which first asked participants whether they were interested in having a child in the future (yes, no, unsure). Those who were *not* interested in family building were asked one follow-up question about reasons for that decision (data reported below) and then were excluded from further questions within the module.

### Measures

**Sociodemographic and clinical variables** included age, race/ethnicity, parenthood status, Ashkenazi Jewish heritage (due to higher rate of *BRCA1/2* gene mutations in this population [19,20]), education, pregnancy history, co-morbid psychiatric diagnoses (anxiety, depression), and degree of relatedness to family member with breast cancer. A binary variable was created to capture history of any preventive cancer-related surgery including prior breast biopsy, mastectomy, oophorectomy, hysterectomy, or salpingectomy.

**Family-building plans** were assessed using a set of investigator-designed items capturing various aspects of decision making. Participants who endorsed a desire for future family building or uncertainty about their plans completed items assessing anticipated timing, intended methods (i.e., natural conception, reproductive medicine that involves testing embryos for genetic mutations, using donor gametes, surrogacy, embryo/oocyte cryopreservation, and adoption or fostering), and the extent to which family history of cancer influenced their family-building plans. When asked about which family-building option they would consider, they could select “yes” or “unsure” for each option.

**Family-building considerations** that may impact reproductive choices and family-building plans were captured by an investigator-designed 22-item list based on literature review and clinical experience from a multidisciplinary team. The list included cancer-related factors (e.g., family history of cancer, concerns about cancer risk, desire to undergo risk reducing surgery), psychosocial factors (e.g., desire for motherhood, love of children, partner’s opinion), cultural factors (e.g., cultural reasons, religious beliefs), and financial factors (e.g., cost of reproductive medicine and adoption, health insurance coverage).

**Perceived knowledge** was measured through an investigator-designed 5-item set of questions asking participants how informed they feel about topics related to genetic risk for cancer and family-building options (i.e., my possible pregnancy risks due to my family history of cancer; my options for cancer screening; the likelihood of passing on a genetic risk for cancer to a child). Participants who selected “does not apply to me” for any item were excluded from the mean composite score prior to analysis. A similar measure has been used in several prior studies and was adapted for this study [21–23]. The measure demonstrated adequate internal reliability in this study (Cronbach’s alpha=.89).

**Decision uncertainty** was measured by the 3-item uncertainty subscale from the Decision Conflict Scale (DCS), adapted to measure perceived uncertainty about family-building decisions. Items refer to the level of uncertainty or “state of mind” regarding a decision (i.e., “I am clear about the best choice for me.”; “I feel sure about what to choose.”; and “This decision is easy for me to make.”). Responses are on a 5-point Likert scale (‘strongly disagree’ to ‘strongly agree’) and were collapsed into three categories for descriptive analyses: agree (strongly agree/agree), neither agree nor disagree, and disagree (strongly disagree/disagree). Mean decision uncertainty scores were calculated and then multiplied by 25, with scores ranging from 0–100 [24]. Internal reliability was adequate (Cronbach’s alpha=.92).

### Data Analysis

Descriptive statistics were used to characterize all variables. Bivariate analyses examined associations between sociodemographic and clinical variables, family-building considerations, and perceived knowledge with decision uncertainty, using Pearson correlations, independent-samples t-tests, and analyses of variance (ANOVAs). Variables associated with decision uncertainty at p<.10 were retained as covariates in multivariable analysis. A multiple linear regression model was specified with decision uncertainty as the dependent variable. Independent variables included covariates (step 1), family-building considerations (step 2), and perceived knowledge (step 3). All analyses used pairwise deletion. Entering perceived knowledge in the final step allowed us to assess its unique contribution to decision uncertainty after accounting for all other variables.

## Results

Sociodemographic characteristics for the study sample (N= 471) are reported in Table 1. Participants were a mean age of 30.3 years (SD = 6.6;) and predominantly non-Hispanic White (75.1%). Most had completed at least a bachelor’s degree (69.6%), with an additional 24.7% reporting vocational training or some college education. Slightly more than half of the sample was nulliparous (58.6%). Approximately one in six participants (16.6%) identified as Ashkenazi Jewish. Categories describing a family history of breast cancer were not mutually exclusive, with 39.4% of participants reporting a first-degree relative (parent, child, or full sibling) and 66.8% reporting a second-degree relative with in-situ, invasive, or metastatic breast cancer; all participants had at least one family member with a history of breast cancer. Most of the sample did not report a history of anxiety (67.5%) or depression (73.0%). Approximately one in eight participants reported a history of at least one cancer-related procedure, including breast biopsy (8.3%), mastectomy (1.7%), oophorectomy (0.7%), hysterectomy (1.3%), or salpingectomy (0.2%). When asked about family-building intentions, almost half (47.8%) of participants endorsed a desire to have children (or more children) in the future, 16.6% were unsure, and 35.7% said no.

### Family-Building Plans & Family-Building Considerations

Those interested in or unsure about future family building (n=303) were asked to indicate which family-building options they were considering (or would consider) using a six-item, multi-select list with options to indicate “yes” or “unsure” next to each (Figure 1). Most participants considered natural conception (85.8%), with only a small group feeling unsure (7.9%). In contrast, women felt more unsure about other family-building options, including egg freezing or embryo cryopreservation for future use, testing embryos for genetic mutations, use of donated eggs, sperm, or embryos to have a child that is not genetically related, use of a surrogate, and adoption or fostering. Most women said their family history of cancer had not impacted family-building decisions (79.9%), though a subset (16.5%) reported a decrease in their likelihood of having children due to their family cancer history. When asked about factors that were considered important to family-building decisions, women endorsed several health-related concerns, including concerns about a future child’s health (39.7%), passing on a genetic risk of cancer (33.5%), concerns about their own health (39.4%), and desire to undergo cancer risk reducing surgery (16.9%; Figure 2). Financial considerations were also commonly endorsed, including health insurance coverage (39.4%) and costs associated with reproductive medicine (38.7%) and adoption (34.1%).

Separately, among those who indicated no interest in having future children (n=168), the most common reasons were having completed their family-building goals (53%) and no desire for a child (43.5%); followed by financial concerns (24.4%), personal health concerns (22.0%), concerns related to pregnancy (20.8%), and fertility problems/infertility (8.3%). Fewer endorsed cancer-related concerns, including concerns about passing on a genetic cancer risk to a child (8.3%), concerns about their own cancer risk (4.2%), or the need to undergo cancer risk–reducing surgery (3.0%). Open-ended “other” responses (11.9%) reflected a range of considerations, including age-related concern, personal values or preferences (e.g., not wanting the responsibility of parenthood), broader societal concerns (e.g., climate change and perceived global instability), personal or familial health considerations (e.g., managing chronic or heritable mental health conditions), prior reproductive loss or medical complications, and relationship circumstances (e.g., not wanting to raise children while single).

### Perceived Knowledge

The mean composite Perceived Knowledge score was 2.62 (SD=1.04; range=1–5), reflecting moderate levels of perceived knowledge about their genetic risk for cancer and family-building options. Item-level response frequencies characterized unmet information needs (Figure 3). For example, regarding the likelihood of passing on a genetic risk for cancer to a child, 58.5% of women reported feeling *not at all* or *slightly* informed. Likewise, 42.9% of women felt *not at all* or *slightly* informed about their personal genetic risk for cancer and 42.2% were not well informed about options for personal cancer screening.

### Decision Uncertainty

The mean composite Decision Uncertainty score was 37.17 (SD=26.02; range=0–100), suggesting a low to moderate level of uncertainty. Based on item-level response frequencies, between 43–57% of women reported some degree of uncertainty related to family-building decisions (Figure 4).

Bivariate analyses examined associations between decision uncertainty and sociodemographic and clinical characteristics, family-building considerations (values), and perceived knowledge. Greater decision uncertainty was associated with younger age (r=−0.187, p=.001), no history of pregnancy (t=6.91, p<.001), fewer children (r=−0.261, p<.001), and identifying as Ashkenazi Jewish (t=−3.40, p<.001). Women who endorsed the following factors as influencing their family-building decisions (i.e., responding “yes” in a yes/no list of factors) reported *higher* decision uncertainty: concerns about personal health (t =−3.380, p<.001), concerns about a future child’s health (t =−4.01, p<.001), concerns about passing on a genetic risk for cancer (t =−2.701, p=.007), family history of cancer (t=−3.230, p=.001), ability to screen embryos for genetic mutations (t[300]=−2.434, p=.016), ability to choose a child’s sex (t =−2.812, p=.005), cost of reproductive medicine (t =−3.669, p<.001), and cost of adoption (t =−2.544, p=.011). In contrast, several non-medical factors related to *lower* decision uncertainty including: consideration of parents’ opinions (t =3.096, p=.002), religious beliefs (t =2.980, p=.003), love of children (t =3.347, p<.001), and timeline to have children (t =3.930, p<.001). Lower perceived knowledge was associated with higher decision uncertainty (r=−0.175, p=.003). Race/ethnicity, education, history of anxiety or depression, history of cancer risk reducing surgery, and number of first-degree and second-degree relatives with a breast cancer history were not associated with decision uncertainty.

For the primary analysis, 18 variables demonstrated associations at the p<.10 level with decision uncertainty. One variable (“passing on a genetic risk for cancer to a child”) was removed due to high collinearity and conceptual overlap with other family-building considerations. The remaining 17 variables were included in the hierarchical multiple regression model (Table 2). Step 1 explained 17.3% of the variance in decision uncertainty (R^2^=.173) and higher decision uncertainty was associated with identifying as Ashkenazi Jewish (β=.181, p< .001) and having no history of pregnancy (β=−.216, p=.002). In Step 2, family-building considerations were added to the model, improving model fit and increasing the variance explained to 29.2% (R^2^=.292). Those who reported concern about ‘timeline to have children’ reported lower decision uncertainty (β=−.135, p=.021), whereas concern about a future child’s health (β=.164, p=.016) related to higher decision uncertainty. In Step 3, perceived knowledge was added to the model, resulting in a final R^2^ of .305. Greater perceived knowledge was associated with lower decision uncertainty, controlling for other factors (β=−.128, p=.026).

## Discussion

This study examined the ways in which a family history of breast cancer shapes reproductive decision making and family-building choices and identifies areas of unmet need for women representing non-clinical populations. Data from the YWS were analyzed, providing an opportunity to validate qualitative findings in the literature with a large, international sample. Findings suggest that uncertainty about family-building options is prevalent and is shaped by sociodemographic, experiential, and values-based factors. Cancer-related health concerns, along with other factors such as financial barriers, appear to be prominent influences on family-building decisions. Knowledge gaps about genetic cancer risk and reproductive/family-building options are common and predicted greater decision uncertainty. Findings underscore the complexity of making family-building decisions when cancer- and health-related risks are considered and identify subgroups of women that may benefit from support and informational resources.

Among those interested in future family building or uncertain about their plans, the vast majority of women considered natural conception (unassisted reproduction), while substantially fewer endorsed — and many more were unsure about — alternative methods such as the use of reproductive medicine including genetic testing of embryos. Likewise, when asked about the overall decision-making process, 43–57% of women endorsed some degree of uncertainty with respect to decision clarity, confidence, and ease of decision making (item-level analysis). A meaningful subgroup of women (16.5%) reported a decrease in their likelihood of having children due to their family history of cancer, along with concerns about passing on a genetic risk for cancer to a child (33.5%). This is consistent with the literature reporting that women with pathogenic genetic variants in cancer susceptibility genes, such as in *BRCA1/2*, report greater uncertainty about having children due to cancer-related fears [25] and changes in family-building plans [26]. The high proportion of women who were unsure about family-building options, coupled with evidence of low perceived knowledge (e.g., nearly 60% feeling uninformed about the likelihood of passing on a genetic risk for cancer to a child), suggests many women worry about cancer-related risks but lack sufficient information relevant to decision making. Awareness of PGT may help alleviate fears about the health of a future child, though women may still worry irrespective of genetic testing results, struggle with decisions about PGT (e.g., moral concerns and necessity of IVF), and may still face barriers to accessing PGT and reproductive medicine (e.g., cost) [27–29]. These findings underscore the need for support that is not only medically informed, but attuned to the personal, relational, and values-based dimensions of family-building decisions (and those of a partner or loved ones, if applicable).

It is important to note that most women (83.5%) reported no decrease in their likelihood of having children, though findings suggest they may still struggle with unmet information needs and decisional uncertainty. For example, 88% of women felt uninformed about possible pregnancy risks due to their family history of cancer. It is well established that women around the world have inadequate knowledge of reproductive health generally and cancer-related concerns may add to uncertainty [30]. Obstetrician-gynecologists and primary care providers may be best positioned to offer counseling, though evidence suggests there are barriers to discussing cancer-related topics within preconception counseling (e.g., lack of time, perceived lack of expertise) [31–33]. Patient and provider educational resources may be developed and effective dissemination strategies should be explored.

Greater uncertainty about family-building decisions was associated with younger age, no prior history of pregnancy, having fewer children, and consideration of family-building timeline. This pattern suggests that women who are earlier in their reproductive life course and those with some sense of reproductive time pressure may struggle more with decisions and planning. Notably, history of pregnancy was the strongest predictor in the final model, suggesting those who have already navigated pregnancy may approach subsequent family-building decisions with more experience, clarity, hope, or confidence. It may also reflect how parenthood intersects with identity and transitions in social roles. Women with no prior history of pregnancy, who do not have children, may represent those with more uncertainty about entering motherhood. Other factors, such as sociocultural beliefs and norms or integrating the opinions of loved ones into decision making, may cause additional uncertainty, perhaps especially for younger women without prior lived experiences as parents [3]. Interestingly, women who endorsed religious beliefs as an important factor related to family-building decisions reported lower levels of decision uncertainty, but those who identified as Ashkenazi Jewish reported higher levels of uncertainty. Sociocultural and religious beliefs clearly influence decisions about reproduction and family building and may be important indicators for when and how to support women who have cancer-related concerns [34,35].

Importantly, financial considerations emerged as a key factor in family-building decisions with nearly 40% of women endorsing costs of reproductive medicine and insurance coverage as influencing factors. Findings underscore system-level structural barriers that shape family-building options, decisions, and access. Medical options to reduce genetic risk in offspring, such as IVF with PGT, are often among the most expensive and least equitably accessible [25,36,37]. Racial and ethnic disparities in utilization of ART and clinical outcomes are well documented and persist even with insurance mandates [38]. Financial concerns are associated with greater decision uncertainty among young adult cancer survivors considering family-building options [39,40]. Further research is needed to characterize the experiences and needs of women with more limited access to healthcare systems and financial resources and the ways in which reproductive choices and family-building experiences are shaped by these factors.

Additionally, a variety of other considerations were endorsed as being relevant and important to family-building decisions, and many of these were significantly related to decisional uncertainty. This is consistent with health decision frameworks in which values-based factors are central to decisional processes [41]. That is, decision uncertainty and distress stem from inadequate knowledge or access to information, unclear values, and inadequate support and resources [42]. Family-building decisions are inherently preference-sensitive; there is no right or wrong choice and people must weigh the risks and benefits of options in the context of personal values, priorities, and goals [43].

Not surprisingly, findings suggest that knowledge is a key target for intervention but may be insufficient for addressing the difficulties women face when considering family-building options amidst cancer-related concerns and other personally relevant factors. Psychoeducation is most effective when delivered alongside opportunities for values exploration [44,45]. Decision aids are often used to support both knowledge acquisition and values clarification, which may be used as adjuncts to clinical care and individualized support [46,47]. Decision aids have been used in women with a predisposition to cancer who haven’t had the disease, (e.g., ePOWER [48]) and among cancer survivors making family-building decisions [49]. They can also be adapted for couples and dyadic support [50,51].

To our knowledge, this study is the first large-scale quantitative investigation of family-building decision making among individuals with a familial risk for breast cancer. While smaller qualitative studies have explored this experience, and more targeted work has examined family-building among those with pathogenic variants in *BRCA1/2* or other genes, quantitative data remain limited. Several limitations should be noted. The cross-sectional design precludes causal inference. Although the sample was international, which is a strength, women were predominantly non-Hispanic White and highly educated. Not all participants completed the Reproductive Choices module due to survey design and desire to minimize participant burden; this module was skipped for participants who reported no interest in future children to ensure questions were appropriate to the population. Measures of perceived knowledge and family-building considerations were investigator-designed and, while demonstrating adequate internal reliability, have not been independently validated. Analyses were not stratified by pathogenic variant carrier status, and it is unknown whether findings may differ based on perceived vs. known genetic risk (e.g., breast cancer family history vs. genetic testing results).

## Conclusion

This study characterized the ways in which women with a family history of breast cancer considered their family-building options, experiences of decision uncertainty, and factors deemed important when making these decisions. Findings indicate that many women feel uninformed about cancer-related risks and family-building options and may struggle to integrate information with values-based considerations. Effective decision support should provide personalized health information while facilitating values clarification, incorporating prior experiences, sociocultural and religious beliefs and values, and broader life context and relationships. Further research is needed to investigate the role of financial stress and access to care, as structural constraints inevitably shape decision-making processes and outcomes.

## Figures and Tables

**Figure 1.**
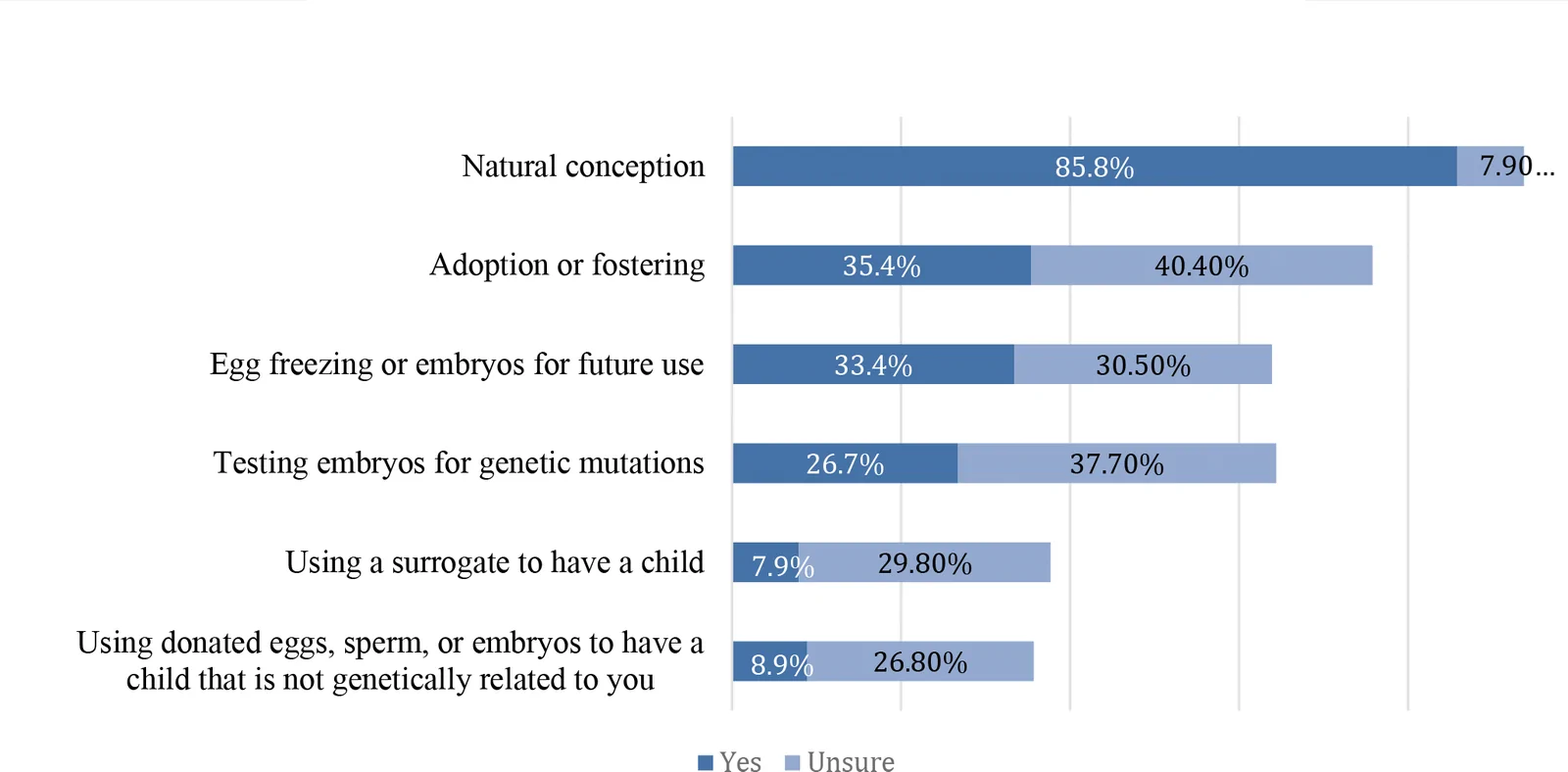
Family-Building Options Being Considered (n=303) Women who reported a desire for future family building or uncertainty about family-building plans were asked to indicate which family-building options they were considering using a multi-select list with options to indicate “yes” or “unsure.”

**Figure 2.**
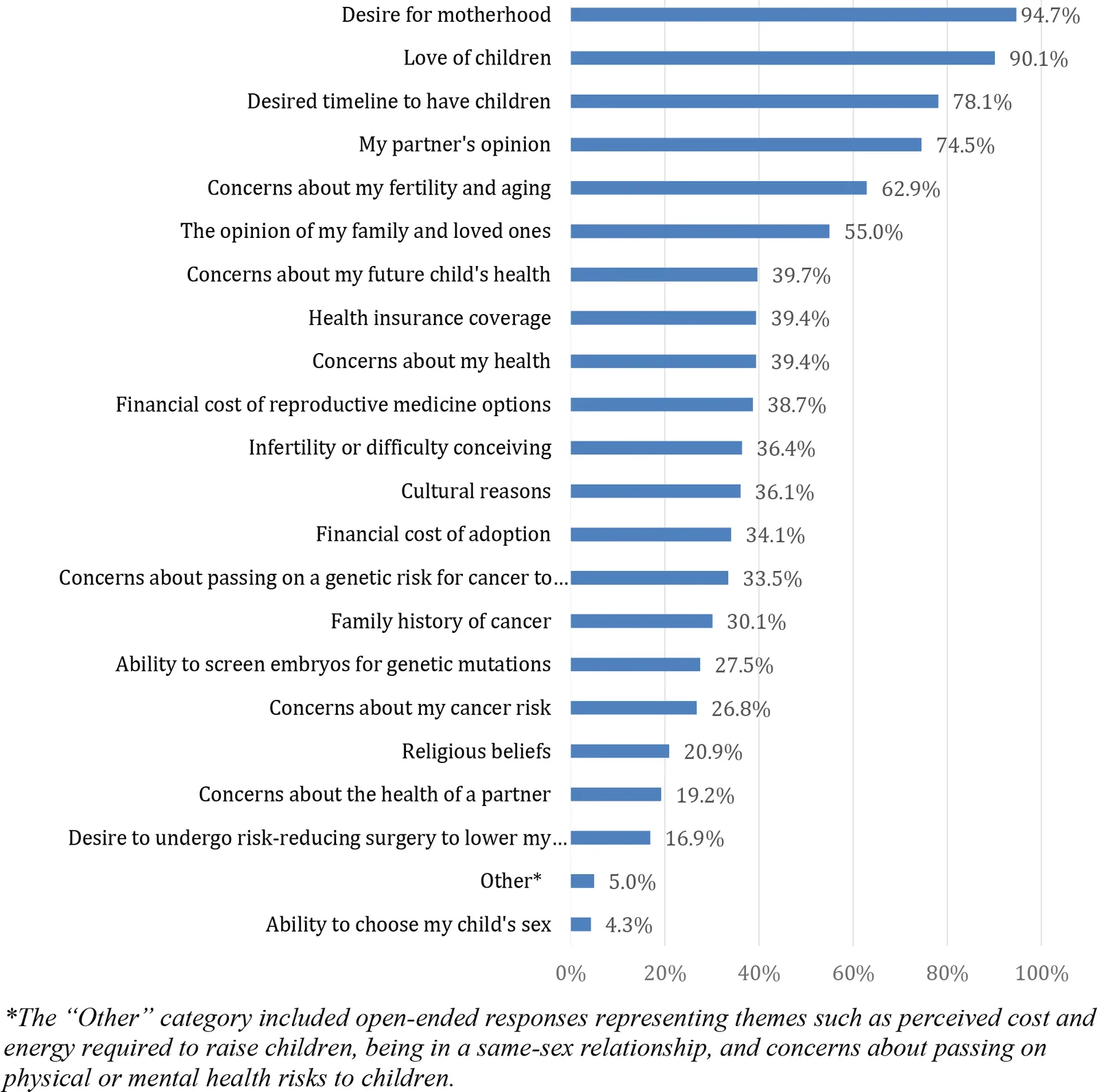
Factors Influencing Family-Building Decisions Among Women Who Want or Might Want Children (n=303) Women who reported a desire for future family building or uncertainty about family-building plans were asked to indicate which factors were influencing their family-building decisions. ^*^The “Other” category included open-ended responses representing themes such as perceived cost and energy required to raise children, being in a same-sex relationship, and concerns about passing on physical or mental health risks to children.

**Figure 3.**
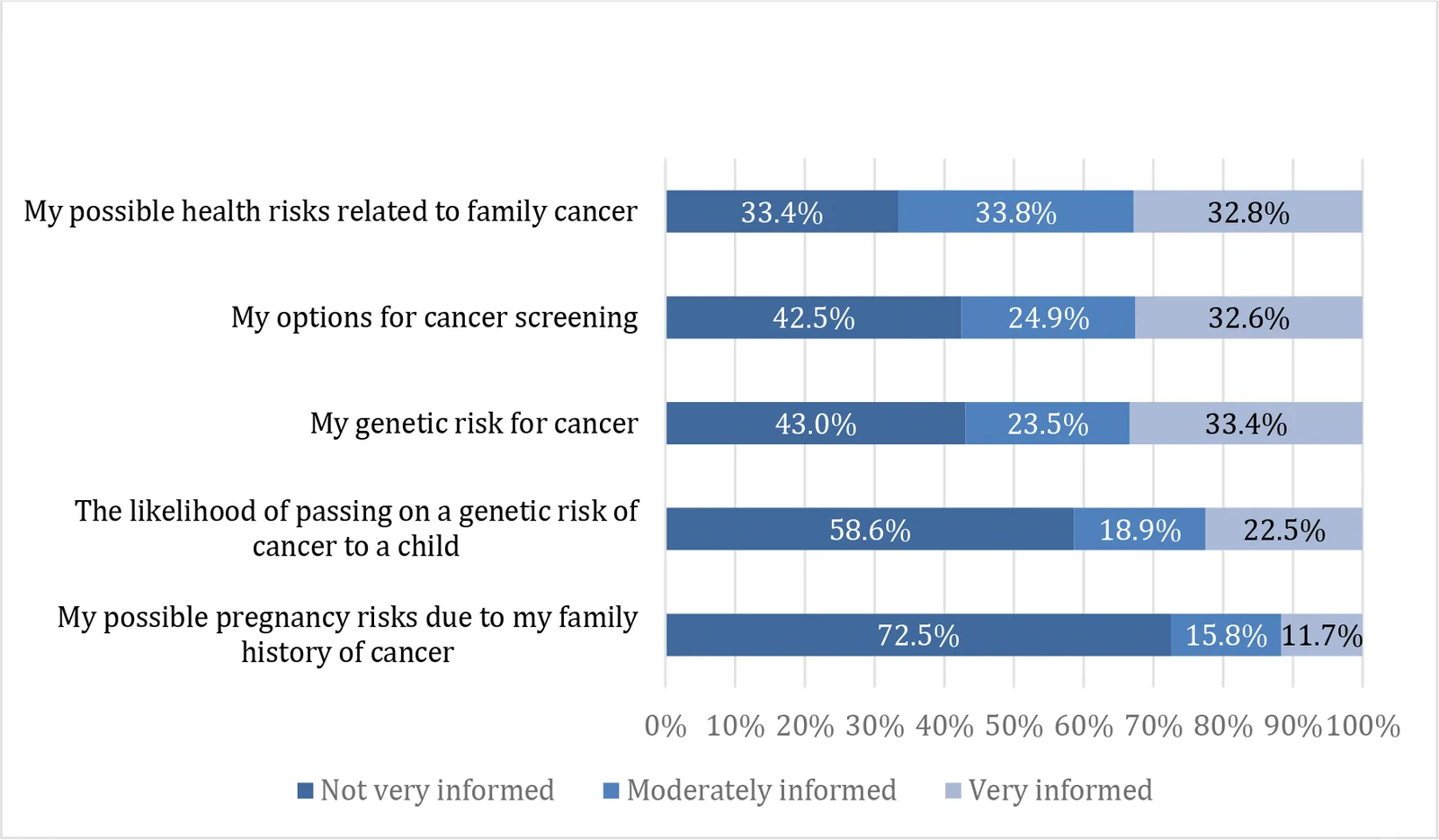
Perceived knowledge about impact of familial cancer on personal health and family-building options (n=303) Women who reported a desire for future family building or uncertainty about family-building plans were asked to answer how informed they felt about these topics.

**Figure 4.**
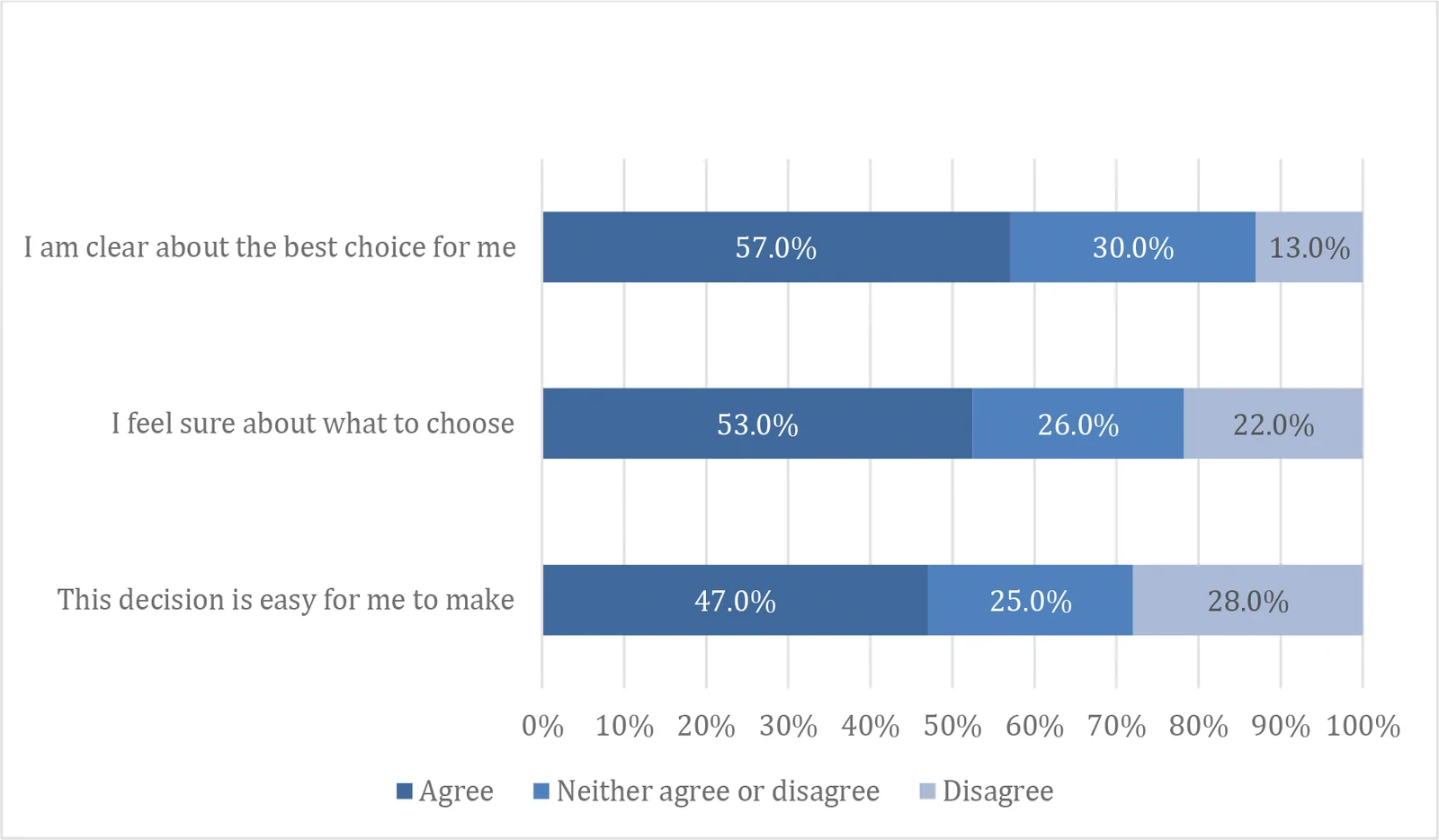
Decision uncertainty about family-building options: item-level responses (n=303) Women who reported a desire for future family building or uncertainty about family-building plans were asked to consider the following aspects of decision regarding family-building decisions.

**Table 1.** Characteristics of the study sample (n=471)

		Mean (SD)	Range
Age (years; at time of completing survey)		30.34 (SD: 6.69)	18 – 45

		Percentage	n
Race/ethnicity	White, non-Hispanic	75.1%	353
	Black, non-Hispanic	1.9%	9
	Hispanic	7.4%	35
	Asian, non-Hispanic	5.7%	27
	Other	9.8	46

Education	≤High school graduation / G.E.D.	5.7%	27
	Vocational or technical school / Some college or university	24.7%	116
	Bachelor’s degree, Graduate degree	69.6%	327

Number of children (< 18)	Nulliparous	58.6%	276
	1	15.5%	73
	2	14.2%	67
	3	6.6%	31
	4+	5.1%	24

Ashkenazi Jewish	Yes	16.6%	78
	No	77%	362
	Other	6.4%	30

Number of first-degree family members with breast cancer history	0	60.6%	243
	1	39.4%	158

Number of second-degree family members with breast cancer history (#)	0	33.2%	133
	1	44.6%	179
	2	16.0%	64
	3	6.0%	24
	4	.2%	1

Anxiety Disorder	Yes	28.7%	135
	No	67.5%	318
	Other	3.8%	18

Clinical Depression	Yes	23.8%	112
	No	73.0%	344
	Other	3.2%	15

History of risk-reducing surgery related to cancer	No	87.5%	400
	Yes	12.5%	57

Hope to have a child (or more children) in the future	Yes	47.8%	225
	Maybe	16.6%	78
	No	35.7%	168

**Table 2.** Multivariable Model Predicting Decision Uncertainty (n=289)

Step	Predictor*	R^2^	B	SE	β	t	p

**1**	Constant	.173	3.551	.385		9.225	<.001
	Ashkenazi Jewish		12.276	3.550	.181	3.458	<.001
	Age (years)		.004	.275	.001	.015	.988
	Number of children		−1.236	1.743	−.043	−.709	.479
	Pregnant Ever		−12.326	3.915	−.216	−3.149	.002

**2**	Timeline to Have Children	.292	−8.495	3.671	−.135	−2.314	.021
	Parents’ Opinion		−1.536	3.421	−.026	−.449	.654
	Religious Beliefs		−4.917	3.448	−.079	−1.426	.155
	Concerns About My Health		1.252	3.296	.024	.380	.704
	Concerns About my Future Child’s Health		8.603	3.535	.164	2.433	.016
	Ability to Choose Child’s Sex		12.652	6.846	.098	1.848	.066
	Ability to Screen Embryos for Genetic Mutations		−3.077	3.533	−.054	−.871	.385
	Cost of Reproductive Medicine		4.595	3.553	.087	1.293	.197
	Cost of Adoption		1.229	3.391	.023	.362	.717
	Love of Children		−8.467	5.100	−.094	−1.660	.098
	Desire for Motherhood		−8.638	7.571	−.064	−1.141	.255
	Family History of Cancer		4.988	3.817	.090	1.307	.192

**3**	Perceived Knowledge	.305	−3.186	1.419	−.128	−2.245	.026

* Collinearity diagnostics indicated no problematic multicollinearity among predictors (VIFs ranged from 1.01 to 1.83), suggesting that correlations among independent variables did not meaningfully affect model estimation.

## Data Availability

The data used in this study were obtained from the Breast Cancer Family Registry (BCFR) Young Women’s Study. Data are not publicly available due to participant privacy and consent restrictions, but qualified researchers may request access through the BCFR data access process. Further information is available at https://www.bcfamilyregistry.org or by contacting the BCFR Data Coordinating Center.
